# Red Blood Cell, White Blood Cell, and Platelet Counts as Differentiating Factors in Cardiovascular Patients with and Without Current Myocardial Infarction

**DOI:** 10.3390/ijms26125736

**Published:** 2025-06-15

**Authors:** Joanna Kostanek, Kamil Karolczak, Wiktor Kuliczkowski, Cezary Watala

**Affiliations:** 1 Department of Haemostatic Disorders, Medical University of Lodz, 6/8 Mazowiecka Street, 92-215 Lodz, Poland; kamil.karolczak@umed.lodz.pl (K.K.); cezary.watala@umed.lodz.pl (C.W.); 2 Institute for Heart Diseases, Wroclaw Medical University, 213 Borowska Street, 50-556 Wroclaw, Poland; wiktor.kuliczkowski@umed.wroc.pl

**Keywords:** myocardial infarction, cardiovascular diseases, blood morphology, bootstrap, resampling procedures

## Abstract

Cardiovascular diseases continue to pose a major global health burden, contributing significantly to mortality rates worldwide. This study aimed to explore the association between myocardial infarction and basic hematological parameters—red blood cells (RBCs), white blood cells (WBCs), and platelets (PLTs)—which are routinely assessed in clinical diagnostics. The analysis was conducted on a cohort of 743 adults hospitalized with diagnosed cardiovascular conditions. To identify blood parameters that distinguish patients with a history of first-time myocardial infarction from those who had never experienced such an event, we employed a dual analytic approach. Standard parametric methods were complemented with bootstrap resampling to strengthen inference and mitigate the impact of sampling variability. Patients with myocardial infarction showed decreased RBC and elevated WBC counts relative to those without infarction. These associations were non-linear, with the most pronounced group differences observed within the second and third quartiles of RBC and WBC distributions, while minimal differences appeared at the distributional extremes. No significant differences were found in platelet count (PLT) between the groups. Bootstrap validation not only corroborated findings obtained through traditional statistics, but also enhanced the robustness of the results, providing improved estimates under data conditions prone to skewness or small sample artifacts. This approach enabled the detection of nuanced patterns that might elude classical inference. Our findings emphasize the utility of resampling techniques in clinical research settings, particularly where inference stability is critical. Incorporating such methods in future investigations may advance statistical rigor, increase reproducibility, and better capture complex biological relationships in medical datasets.

## 1. Introduction

Cardiovascular diseases have remained a leading global health challenge for decades, causing the deaths of millions worldwide each year [1]. Despite notable progress in medicine and the development of advanced diagnostic tools, both their prevalence and associated mortality remain persistently high [2]. Lifestyle-related factors—such as tobacco use, insufficient physical activity, and excessive alcohol consumption—are widely recognized as major contributors to cardiovascular risk. Furthermore, comorbid conditions including hypertension, diabetes, and obesity, as well as biochemical blood parameters such as lipid profiles and glucose metabolism indicators, are critical for accurate risk assessment [3].

In routine clinical practice, basic hematological parameters such as red blood cells (RBCs), white blood cells (WBCs), and platelets (PLTs) and the results of standard biochemical tests often constitute the first available diagnostic information—even before more specialized cardiological investigations are performed [4]. Despite their widespread use, the extent to which peripheral blood counts can accurately distinguish patients at high cardiovascular risk from those at low risk or differentiate individuals with a history of myocardial infarction from those without such events remains insufficiently understood.

Platelets play a pivotal role in the pathogenesis of atherosclerosis; thus, their morphological and functional characteristics may be regarded as critical components in cardiovascular risk assessment [5]. Excessive platelet reactivity is considered one of the principal factors increasing the likelihood of major cardiovascular events [6], but the analysis of platelet indices within routine laboratory diagnostics of cardiological patients, especially those with myocardial infarction, is still limited.

Similarly, the recognition of chronic inflammation as a key contributor to atherosclerosis and other cardiovascular diseases has sparked growing interest in its reflection in hematological variables [7]. Particular attention has been directed toward WBC count as a potential marker of cardiovascular risk. Emerging evidence suggests that elevated leukocyte levels may correlate with an increased risk of coronary events, including myocardial infarction, underscoring the importance of incorporating this parameter into cardiovascular risk assessment models [8].

Perhaps most intriguing is the potential role of red blood cells as either pathogenic or protective factors in cardiovascular disease, particularly under conditions that may predispose to myocardial infarction. As the most abundant blood cells, erythrocytes contribute significantly to blood viscosity—an established physical property associated with atherogenesis [9]. On the other hand, emerging evidence suggests that red blood cells may serve as carriers of nitric oxide synthase, thereby contributing to the production of nitric oxide, a critical vasodilatory molecule [10]. Furthermore, their primary function—oxygen transport—clearly has implications for myocardial oxygenation. This duality of erythrocyte function stems from their opposing effects on cardiovascular health. On one hand, an increased RBC count elevates blood viscosity, leading to thicker blood and impaired flow, which consequently may increase the risk of vascular occlusion [11]. On the other hand, RBCs exert protective effects by facilitating oxygen transport, enhancing tissue oxygenation, and serving as carriers of nitric oxide synthase—the enzyme responsible for the production of nitric oxide (NO), a potent vasodilator that maintains endothelial function and vascular homeostasis [12]. This complex interplay between detrimental and protective mechanisms positions erythrocytes as a particularly compelling focus of the present analysis.

Thus, the aim of this analysis is to identify hematological variables (RBC, WBC, PLT) that significantly differentiate cardiovascular patients who have experienced a first myocardial infarction from those who have never suffered such an event. These variables were selected based on their established links to key pathophysiological mechanisms, and RBCs seem to have the most dual effect among them, with respect to their seeming opposing impacts. While WBCs and PLTs act clearly in a specific direction (WBC counts reflect the degree of systemic inflammation involved in atherogenesis, and PLT counts are critical indicators of thrombotic risk), the impact of RBC levels demonstrates a peculiar duality: they aggravate blood viscosity on one hand and improve tissue oxygen transport on the other. In this study, two complementary analytical approaches were employed. The first involved conventional statistical methods. The second utilized the bootstrap technique to provide an additional layer of inference validation beyond the classical framework.

Originally introduced by Bradley Efron, bootstrapping is a resampling-based method that relies on repeated sampling with replacement from the original data [13]. It enables estimation of sampling distribution without the need to assume any specific population distribution. Owing to its flexibility, the bootstrap approach facilitates the application of statistical procedures that might otherwise be inappropriate under classical assumptions. Incorporating the bootstrap method thus enhances the robustness and reliability of analytical findings.

In our study, we examined the relationship between myocardial infarction and hematological variables, both in the overall patient population and in subgroup analyses based on quartile stratification by RBC and WBC counts. This stratified approach enabled a more nuanced understanding of how variations in these parameters may be associated with the occurrence of myocardial infarction across different patient subgroups. Importantly, quartile-based grouping allowed us to account for clinical heterogeneity within the studied cohort, thereby yielding more granular insights into the associations between morphological markers and infarction risk—insights that may inform future research directions.

Although the initial sample size was relatively large, stratification into quartiles substantially reduced the number of observations in some subgroups, potentially introducing instability or bias to classical statistical estimates. To address this limitation, the bootstrap method was applied. By mitigating the impact of random fluctuations arising from small subgroup sizes, this approach enhanced the reliability of statistical inference and enabled more precise evaluation of the relationships between hematological markers and myocardial infarction occurrence.

## 2. Results

### 2.1. Dataset Characteristics

The analysis included data from 743 adult patients hospitalized at the Department of Cardiology, Wroclaw Medical University, between 2015 and 2018, all of whom were diagnosed with cardiovascular disease. Among them, 620 patients had no history of myocardial infarction, while 123 individuals were admitted due to a recent first-time myocardial infarction. The basic clinical characteristics of the study population are presented in Table 1. Comprehensive information regarding the prevalence of cardiovascular conditions and comorbidities is provided in the Supplementary Materials (Supplementary Data, Table S1).

### 2.2. Interdependence Analysis and Variable Selection for Logistic Regression Modeling Based on Correlations Between Peripheral Blood Parameters in Cardiovascular Patients

**Figure 1:**
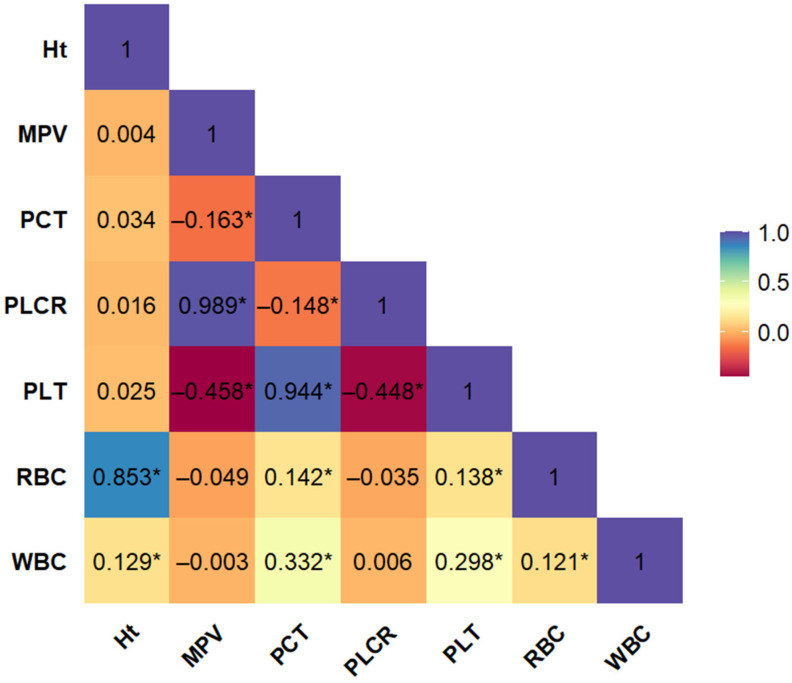
Bootstrap-boosted linear Pearson’s correlation plot of morphological variables in the whole population of patients with cardiovascular disease (*n* = 743). The values represent the bootstrap-boosted linear Pearson’s correlation coefficient (10,000 iterations). Statistically significant correlations (*p* < 0.05) are marked with an asterisk (*). Abbreviations: Ht = hematocrit, MPV = mean platelet volume, PCT = plateletcrit, PLCR = platelet–large cell ratio, PLT = number of blood platelets, RBC = number of red blood cells, WBC = number of white blood cells.

The aim of the preliminary phase of the analysis was to assess differences in routinely measured hematological parameters between cardiovascular patients with and without a history of myocardial infarction (MI(−) and MI(+), respectively). Therefore, prior to performing the core logistic regression analysis, we evaluated the interdependence among the hematological variables. Pairwise, unadjusted correlation coefficients were calculated for all morphological blood parameters (Figure 1). The analysis revealed notable multicollinearity among several variables. Red blood cell count (RBC) showed a strong positive correlation with hematocrit (Ht), while platelet count (PLT) was highly correlated with mean platelet volume (MPV), plateletcrit (PCT), and platelet large cell ratio (PLCR). Due to this high degree of intercorrelation, the variables Ht, MPV, PCT, and PLCR were excluded from further modeling to avoid redundancy and multicollinearity-related bias. Consequently, only RBC, WBC, and PLT were retained for subsequent stages of analysis.

### 2.3. Differences in Leukocyte, Erythrocyte, and Platelet Counts in Cardiovascular Patients with Current and First-Time Myocardial Infarction Compared to Those Without a History of Infarction

**Figure 2:**
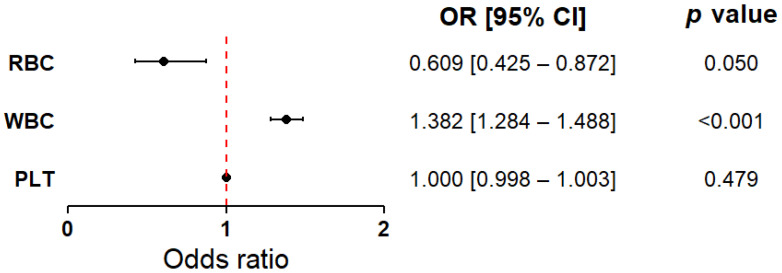
Forest plot showing the odds ratios for white blood cell count, red blood cell count, and platelet count as discriminative factors between cardiovascular patients with current, first-time myocardial infarction and those without a history of infarction. The results are presented as odds ratios (ORs) with 95% confidence intervals (CIs), calculated using bootstrap resampling (10,000 iterations). Circles represent point estimates of the ORs, and error bars indicate 95% CIs. The dashed vertical line denotes the null effect value (OR = 1.0), indicating no increased or decreased risk. The analysis was conducted in the total study population (MI(−) and MI(+) combined, *n* = 743), with group sizes adjusted to 372 patients per group (see Materials and Methods). The model was adjusted for age and sex. Based on the Hosmer–Lemeshow test (*p* > 0.05), the model was considered to have a good fit. Abbreviations: PLT—platelet count; RBC—red blood cell count; WBC—white blood cell count.

Figure 2 presents the results of logistic regression analyses performed for the key hematological variables (RBC, WBC, PLT). The models were adjusted for age and sex as covariates. A higher white blood cell count (WBC) was significantly associated with the presence of myocardial infarction compared to the control group (OR = 1.38, 95% CI: 1.28–1.49, *p* < 0.001). Conversely, red blood cell count (RBC) was lower in patients with myocardial infarction compared to those without (OR = 0.61, 95% CI: 0.42–0.87, *p* = 0.050). Although the *p*-value was at the threshold of statistical significance (*p* < 0.05), the observed effect suggested a potentially meaningful role for RBC in differentiating between patient groups. Accordingly, RBC was retained for further analyses. No significant differences were found in platelet count (PLT) between the groups (OR = 1.00, 95% CI: 0.998–1.003, *p* = 0.479). Due to its neutral effect and lack of statistical association, PLT was excluded from subsequent stages of the analysis. The results, including both classical and bootstrap-validated models, as well as detailed information regarding the control variables (age and sex), are presented in the Supplementary Materials (Supplementary Data, Table S3). To complement these findings, separate logistic regression models were constructed using alternative platelet-related indices available in the dataset, including PCT, MPV, and PLCR. However, none of the evaluated platelet indices showed statistically significant associations with the presence of myocardial infarction in the total study population (Supplementary Data, Table S4).

In addition, a stratified analysis was conducted based on myocardial infarction subtypes—STEMI and NSTEMI—to explore potential differences in the evaluated hematological markers. In both subgroups, RBC levels were significantly lower, and WBC levels significantly higher, compared to the control group (MI-). No significant differences in PLT were observed for either infarction subtype. The lack of substantial differences between STEMI and NSTEMI in terms of the relevance of RBC and WBC to infarction occurrence supported the decision to continue further analyses using a combined group of patients with myocardial infarction. Detailed comparative data between STEMI and NSTEMI are provided in the Supplementary Materials (Supplementary Data, Table S3).

### 2.4. Association Between Red Blood Cell Count (RBC) and Myocardial Infarction—Quartile-Based Analysis

To more precisely determine the range in which red blood cell count (RBC) most effectively differentiates between patients with myocardial infarction (MI(+)) and those without (MI(−)), a logistic regression analysis was conducted within RBC quartile subgroups. Two models were evaluated—one including RBC alone and another combining RBC with white blood cell count (WBC)—to assess potential interactive or cumulative effects of these parameters. Table 2 summarizes the clinical characteristics of the study population stratified by RBC quartiles (Q1–Q4). Patients in the lowest quartile (Q1) had the highest mean age and the greatest proportion of women. In contrast, the highest quartile (Q4) was predominantly composed of men and had the lowest mean age among all quartiles. Mean WBC and platelet (PLT) counts remained relatively stable across RBC quartiles. In all quartile groups, the number of patients without myocardial infarction exceeded those with MI, and the proportions were relatively consistent across quartiles.

**Figure 3:**
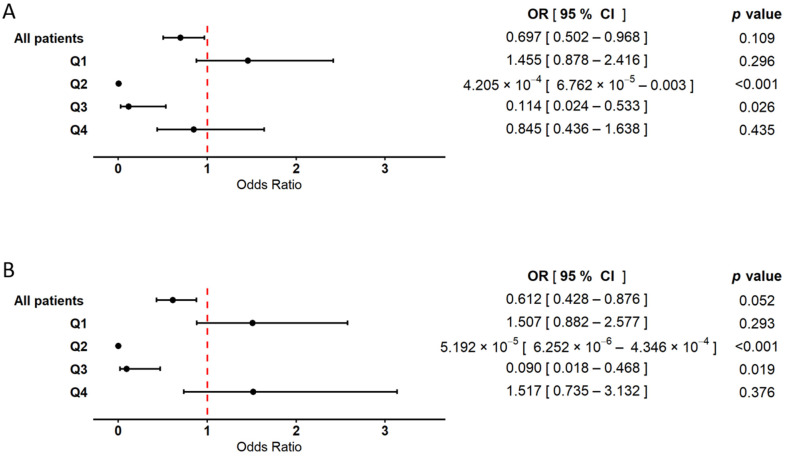
Forest plot illustrating the association between red blood cell count (RBC) and the occurrence of myocardial infarction. The results are based on logistic regression models with bootstrap resampling. Odds ratios (ORs) and 95% confidence intervals (CIs) were calculated using 10,000 bootstrap iterations, with sample sizes adjusted to 372 patients in both the MI(+) and MI(−) groups. Circles represent point estimates of ORs, and error bars indicate 95% CIs. The dashed vertical line marks the null effect threshold (OR = 1.0), indicating no harmful or protective effect. The analysis was performed in two models: (**A**) a model including RBC as the explanatory variable, adjusted for age and sex, and (**B**) a model including both RBC and WBC as explanatory variables, also adjusted for age and sex. In both models, analyses were conducted for the overall patient cohort, as well as within RBC quartile subgroups (Q1–Q4). The Hosmer–Lemeshow goodness of fit test yielded *p*-values greater than 0.05 for all models, indicating good model fit.

Logistic regression analysis including RBC only (Figure 3A) revealed statistically significant associations in quartiles Q2 and Q3—lower red blood cell counts were more frequently observed among MI(+) patients compared to those without myocardial infarction (MI(−)). A similar inverse relationship was observed in the overall cohort; however, the effect did not reach statistical significance. In the extreme quartiles (Q1 and Q4), no statistically significant associations were found. In the model incorporating both RBC and WBC (Figure 3B), the statistically significant inverse associations between RBC and MI in Q2 and Q3 remained consistent. In the total patient population, the relationship between RBC and MI was weaker and not statistically significant. Again, no significant associations were observed in Q1 and Q4. The results from both the classical statistical approach and bootstrap validation, as well as detailed information regarding the control variables (age and sex), are provided in the Supplementary Materials (Supplementary Data, Table S5).

### 2.5. Association Between White Blood Cell Count (WBC) and Myocardial Infarction—Quartile-Based Analysis

To evaluate the role of white blood cell count (WBC) as a distinguishing factor between MI(+) and MI(−) patients, a quartile-based logistic regression analysis was conducted. As with RBC, two models were analyzed––one including WBC alone and the other including both WBC and RBC––to assess potential interactions between these variables. The basic characteristics of all variables across WBC quartiles are presented in Table 3. Patients in the highest quartile (Q4) had the lowest mean age, whereas the highest mean age was observed in quartile Q2. The most pronounced sex differences were noted in Q3 and Q4, where the proportion of male patients exceeded that of females. Mean RBC and platelet (PLT) counts were lowest in Q1 and highest in Q4. The lowest proportion of patients with myocardial infarction was observed in Q1, while the highest was found in Q4.

**Figure 4:**
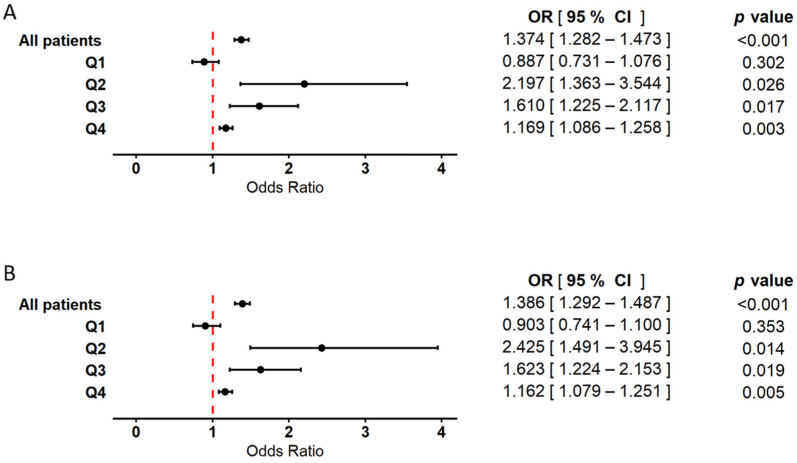
Forest plot illustrating the association between white blood cell count (WBC) and the occurrence of myocardial infarction. Results are based on logistic regression models with bootstrap resampling. Odds ratios (ORs) and 95% confidence intervals (CIs) were calculated using 10,000 bootstrap iterations, with group sizes adjusted to 372 patients in both the MI(+) and MI(−) cohorts. Circles represent point estimates of ORs, and error bars indicate 95% CIs. The dashed vertical line denotes the null effect threshold (OR = 1.0), indicating no harmful or protective association. The analysis was conducted using two models: (**A**) a model including WBC as the explanatory variable, adjusted for age and sex, and (**B**) a model including both RBC and WBC as explanatory variables, also adjusted for age and sex. In both models, analyses were performed in the overall population, as well as within subgroups stratified by WBC quartiles (Q1–Q4). Hosmer–Lemeshow test values exceeded 0.05 in all models, indicating good model fit.

Logistic regression analysis conducted within the WBC quartile subgroups revealed primarily positive associations between WBC count and myocardial infarction (Figure 4). In the model including WBC alone (Figure 4A), odds ratios (ORs) were significantly elevated in quartiles Q2 and Q3. In quartile Q4, a moderately increased OR was also observed and reached statistical significance, although the effect was less pronounced compared to Q2 and Q3. In contrast, the association between WBC and MI in Q1 did not reach statistical significance. In the model incorporating both WBC and RBC (Figure 4B), the positive association between WBC count and myocardial infarction remained consistent. The ORs in Q2 and Q3 were statistically significant, while Q4 also showed a significant, though less prominent, association. No statistically significant relationship was found in Q1. In the analysis of the total patient cohort, WBC count demonstrated a significant positive association with myocardial infarction in both models, with comparable OR values. The results from both the classical and bootstrap-validated approaches, along with details regarding the control variables (age and sex), are provided in the Supplementary Materials (Supplementary Data, Table S6).

## 3. Discussion

Among the hematological markers analyzed (RBC, WBC, and PLT), only RBC and WBC demonstrated statistically significant differences between groups. Platelet count, despite its well-established role in the pathogenesis of atherosclerosis, did not differ significantly between MI(+) and MI(−) patients and was therefore excluded from further stages of analysis. Nevertheless, our findings do not rule out a potential role for platelets in the development of myocardial infarction; rather, they suggest that platelet count alone may be insufficient as a discriminatory factor. Current research indicates that platelet function—such as increased reactivity, adhesion and aggregation potential, and participation in inflammatory cascades—may be more relevant to the onset of coronary events than absolute platelet number [5,6].

In the entire cohort of cardiovascular patients analyzed in this study (including both MI(−) and MI(+) individuals), RBC was inversely associated with the occurrence of first-time myocardial infarction. However, this association was at the threshold of statistical significance, indicating the possibility of a more complex, non-linear relationship between RBC and MI. This finding prompted a quartile-based analysis to further investigate the nature of this association.

Within RBC quartile subgroups, the results confirmed the non-linear pattern of association: statistically significant effects were observed in quartiles Q2 and Q3, while no significant differences were noted in the extreme quartiles Q1 and Q4. Importantly, when WBC count was added to the logistic regression model, the effect of RBC was slightly attenuated in some quartiles. This suggests potential interdependence between RBC and WBC in relation to MI risk, with possible offsetting effects.

These findings indicate that RBC may serve as a discriminating factor between MI(+) and MI(−) patients, primarily within the mid-range of values. Therefore, it can be hypothesized that, for patients with RBC values at the lower and upper ends of the reference range, RBC may have limited utility in distinguishing between MI(−) and MI(+) cases, estimating the risk of first-time infarction in cardiovascular populations, or assessing the effectiveness of cardioprotective preventive strategies. These hypotheses warrant further investigation in future targeted studies.

In our study, significant associations between RBC and myocardial infarction were observed exclusively in the middle quartiles (Q2 and Q3), where the odds ratios (ORs) indicated an inverse relationship between RBC and MI among patients with cardiovascular disease (CVD). In contrast, in the lowest quartile (Q1), higher RBC levels were associated with myocardial infarction, although this association did not reach statistical significance. A similar pattern was reported by A.N. Kul et al., who found that patients with acute myocardial infarction (AMI)—both ST-elevation and non-ST-elevation—had significantly lower RBC counts and hematocrit levels (a measure of the proportion of red cells in total blood volume) compared to healthy controls [14]. Our findings are consistent with these observations, suggesting that reduced RBC count may serve as a meaningful marker for distinguishing MI(+) from MI(−) patients. It is important to note, however, that, in our study, the control group consisted of CVD patients without prior infarction, rather than healthy individuals, which may have influenced the observed associations. Nonetheless, both our findings and previous studies point to a link between lower RBC levels and the presence of myocardial infarction. Furthermore, earlier research has shown that reduced RBC count is associated with higher mortality among patients with heart failure, further emphasizing the relevance of RBC as a biomarker in the context of cardiovascular disease [15].

Beyond their primary role in gas transport, RBCs contribute significantly to the regulation of blood rheology and cardiovascular homeostasis [16,17]. They are also involved in the synthesis and release of bioactive forms of nitric oxide (NO), a key mediator of vasodilation. This process is mediated through nitric oxide synthase (NOS) activity within erythrocytes, as well as through interactions between NO and hemoglobin, with these interactions modulated by oxygen tension. NO acts as a potent vasodilator, promoting blood flow and regulating vascular tone [18,19]. As an essential regulator of blood flow and pressure, reduced NO bioavailability has been implicated in the development of atherosclerosis and vascular dysfunction [20,21]. Although the vascular endothelium is traditionally considered the primary source of NO, emerging evidence suggests that RBCs also play an active role in NO metabolism and therefore contribute to the pathophysiology of cardiovascular diseases (CVDs), including myocardial infarction.

Our findings indicate an inverse association between RBC count and the occurrence of myocardial infarction (MI), which may be linked to the role of red blood cells in regulating nitric oxide (NO) bioavailability. As reported in the literature, RBCs can both limit NO availability through reactions with oxyhemoglobin and contribute to its synthesis and release, particularly under hypoxic conditions [22]. In states of reduced RBC count, NO bioavailability may be diminished, potentially promoting endothelial dysfunction and the progression of cardiovascular disease (CVD). Furthermore, in patients with ST-elevation myocardial infarction (STEMI), RBCs may exert cardioprotective effects through NO-dependent signaling pathways, suggesting their involvement in adaptive myocardial defense mechanisms [23]. The proposed mechanism may involve the generation and export of cyclic guanosine monophosphate (cGMP) by erythrocytes in response to hypoxia, exerting a cardioprotective paracrine effect [24].

Our results suggest that the inverse association between RBC count and myocardial infarction (MI) may be non-linear. This negative relationship is most evident in the mid-range quartiles (Q2 and Q3). This finding may indicate that moderately reduced RBC levels within these ranges are associated with impaired regulation of nitric oxide (NO) bioavailability, which could negatively affect endothelial function and promote MI occurrence. Conversely, within the same cardiovascular patient population, higher RBC levels in this range may exert a protective effect.

Although a low RBC count has been associated with increased mortality among patients with cardiovascular disease (CVD), elevated RBC levels may also exert adverse effects. Blood viscosity, which plays a significant role in the course of acute myocardial infarction (AMI), is initially determined primarily by hematocrit. Patients presenting with higher blood viscosity at the onset of infarction are at increased risk of complications [25]. Elevated erythrocyte counts and increased whole-blood viscosity have been linked to a higher risk of ST-elevation myocardial infarction (STEMI) [26]. Furthermore, higher hematocrit levels have been associated with increased mortality from AMI, particularly in younger women [27]. Elevated hematocrit has also been significantly correlated with known CVD risk factors and may serve as a useful diagnostic marker in cardiovascular risk assessment [28]. However, other studies have failed to demonstrate a consistent and statistically significant association between RBC count, hematocrit, and cardiovascular disease [29]. In our study, no significant differences in high RBC levels (Q4) were observed between MI(+) and MI(−) patients within the CVD population. This may reflect the fact that the majority of patients maintained RBC levels within the normal reference range, or that meaningful differences do not exist at the higher end of the distribution among CVD patients with and without myocardial infarction.

Our analysis showed that, in addition to RBC, WBC was a significant factor associated with the occurrence of myocardial infarction. Cardiovascular patients who experienced a first-ever myocardial infarction had significantly higher WBC levels compared to patients with cardiovascular disease but no history of infarction. This conclusion was based on logistic regression performed in the entire study cohort, both in a model adjusted for age and sex and in a model including both RBC and WBC, also adjusted for these covariates.

To more precisely determine the range in which WBC most effectively differentiates between MI(+) and MI(−) patients, a quartile-based analysis was conducted. The results revealed a non-linear association, with the strongest effects observed in quartiles Q2 and Q3. In these subgroups, odds ratios (ORs) were significantly elevated in both models, suggesting that moderately elevated WBC levels may be most relevant for patient differentiation. Interestingly, the effect in the highest quartile (Q4) was also statistically significant, although less pronounced than in Q2 and Q3. The relationship between elevated WBC and the pathogenesis of myocardial infarction may reflect underlying pro-inflammatory mechanisms, as inflammation induces the mobilization and activation of immune cells within the vascular system [30]. Chronic inflammation not only initiates, but also accelerates, the progression of atherosclerosis, which remains a central driver of cardiovascular disease (CVD). Consequently, WBCs may serve as a potential biomarker of cardiovascular risk, reflecting sustained inflammatory activation and its adverse effects on vascular function.

Our findings align with previous research indicating that the WBC count plays a significant role in the pathophysiology of myocardial infarction and cardiovascular disease. Numerous studies have linked elevated WBC levels to increased mortality in the context of CVD. These findings have been comprehensively summarized in a recent review by Park et al. (2023) [31]. Most of the reviewed studies showed that the association between post-infarction survival and WBC count becomes evident primarily at higher WBC levels.

Elevated WBC count has also previously been associated with increased in-hospital mortality among patients with acute myocardial infarction, with the highest mortality observed in individuals with WBC values exceeding the reference range [32]. Moreover, this correlation is not limited to survival outcomes—higher WBC counts have also been linked to greater infarct size, as assessed by myocardial injury biomarkers such as creatine kinase and troponin [33]. These findings are consistent with previous evidence indicating that WBC count is an independent prognostic factor for mortality and may aid in risk stratification following acute myocardial infarction (AMI) [34]. Additionally, elevated WBC levels above the reference range appear particularly relevant in STEMI patients undergoing primary percutaneous coronary intervention, where higher leukocyte counts have been positively correlated with both increased mortality and infarct size [35].

These observations suggest that WBC may not only reflect a patient’s underlying inflammatory state, but also actively contribute to infarct progression and clinical deterioration. Although our study did not focus on the risk of myocardial infarction onset or post-infarction mortality, it demonstrated that WBC—even within mid-normal laboratory ranges—can differentiate between cardiovascular patients with and without a history of myocardial infarction. This raises the possibility that elevated WBC levels, even when still within the reference range, may serve as a differentiating marker between MI(−) and MI(+) patients, and potentially as a prognostic factor.

While the literature cited and our findings differ in terms of the outcome variables assessed (i.e., post-infarction mortality in relation to WBC versus infarction occurrence and group differentiation based on WBC), a shared conclusion emerges: the undeniable role of inflammation in the pathogenesis of myocardial infarction. These findings collectively point to the importance of reducing systemic inflammation as a key objective of cardioprotective preventive strategies. Moreover, routine cardiological monitoring—including at the level of primary care—should consider inflammation as a critical target for early intervention and risk stratification.

## 4. Materials and Methods

### 4.1. Study Population

The analysis was conducted on anonymized data from 743 adult patients with cardiovascular disease, including individuals experiencing an acute myocardial infarction at the time of enrollment, as well as patients with no prior or current history of this cardiovascular event. All participants were hospitalized at the Department of Cardiology, Wroclaw Medical University, between 2012 and 2015. Patients with values for key morphological variables included in the analysis that deviated substantially—either above or below the reference range—were excluded from the analysis. MI was diagnosed according to the Third Universal Definition of Myocardial Infarction (in effect until 2018). Blood samples were collected after hospital admission but prior to coronary angioplasty or other procedures.

The experiments reported here were carried out in accordance with the ethical principles outlined in the 1975 Declaration of Helsinki for research involving human subjects. All the procedures involving human subjects were approved by the Committee on the Ethics of Research in Human Experimentation at the Medical University of Wroclaw (Approval No. KB-73/2012). A written description of the study—including its objectives, protocol, potential risks, and benefits—was provided to each participant during the recruitment process. Informed written consent was obtained from all participants prior to their inclusion in the study.

### 4.2. Sample Collection

Venous blood samples were obtained from forearm veins in a fasting state, using the BD Vacutainer (Becton Dickinson UK Ltd., Plymouth, UK) collection system with plastic tubes coated internally with spray-dried silica to facilitate serum separation, in accordance with the manufacturer’s protocol. Serum samples, obtained without the use of anticoagulants and intended for routine diagnostic testing (mainly estimation of the serum biochemical parameters reported in Table 1), were centrifuged at 2500× *g* for 15 min at room temperature. The separation of serum was completed within a maximum of 4 h after sample collection.

For hematological analysis (including estimation of RBC, WBC, and PLT) and evaluation of concentration of glycated hemoglobin (HbA1c), whole blood was collected into EDTA-containing tubes (Becton, Dickinson and Company Limited, Ireland).

Total cholesterol (TC), triglycerides (TG), and high-density lipoprotein cholesterol (HDL) were measured using enzymatic colorimetric methods on a fully automated Cobas Integra 400 plus biochemical analyzer (Roche Diagnostics GmbH, Mannheim, Germany), following the manufacturer’s instructions (test IDs: TC 2nd generation—0-586; TG—0-010; HDL 3rd generation—0-331). The concentration of low-density lipoprotein cholesterol (LDL) was calculated using the Friedewald formula LDL [mmol/L] = TC − (HDL + TG/2.2), applicable when TG < 4.5 mmol/L.

High sensitivity C-reactive protein (hsCRP) was determined using a biochemical analyzer (Cobas Integra^®^ 400 plus System from Roche Diagnostics GmbH; test ID: hsCRP, 0-033), in accordance with the manufacturer’s specifications. Glucose concentration was assessed photometrically using the Cobas c system (Roche Diagnostics GmbH) according to the manufacturer’s specifications (Glucose HK 3rd generation, test ID: 05 6831 6).

### 4.3. Statistical Analysis

Descriptive statistics for the analyzed variables were reported as arithmetic means with standard deviations (SDs) for continuous variables and as percentages for categorical variables. Correlations between hematological variables were assessed using Pearson’s correlation coefficient with bootstrap analysis. Logistic regression models were employed to estimate odds ratios (ORs) and corresponding 95% confidence intervals (CIs) for the association between hematological markers and the occurrence of myocardial infarction. Model fit was evaluated using the Hosmer–Lemeshow goodness of fit test, with *p* > 0.05 indicating adequate model calibration. All regression models were adjusted for age and sex as covariates. Bootstrap procedures were performed with 10,000 iterations to obtain stable and reliable estimates.

Sample sizes in both the overall and quartile-based analyses were balanced by including 372 patients with myocardial infarction (MI(+)) and 372 patients without prior infarction (MI(−)). This allocation was based on the total cohort size of 743 individuals and ensured equal group sizes, thereby minimizing class imbalance and improving the robustness of comparative analysis. All statistical analyses were performed using STATISTICA software (Dell Inc., Round Rock, TX, USA, 2016; Dell Statistica, Data Analysis System, version 13; software.dell.com) and the R platform (version 4.4.0 for Windows), incorporating publicly available packages and custom-written scripts. A two-tailed *p*-value < 0.05 was considered statistically significant.

## 5. Conclusions

Cardiovascular patients who experienced a first-time myocardial infarction exhibited lower red blood cell (RBC) counts and higher white blood cell (WBC) counts compared to cardiovascular patients without infarction. The associations between RBC, WBC, and the occurrence of myocardial infarction were found to be non-linear. The most distinct differentiation between MI(−) and MI(+) patients was observed in the middle quartiles (Q2 and Q3) of RBC and WBC values, while no significant discrimination was noted in the extreme quartiles (Q1 and Q4 for RBC, and Q1 for WBC). Based on the existing literature, the associations observed with RBC may relate to reduced nitric oxide (NO) bioavailability due to a lower number of NO-releasing erythrocytes in the cardiovascular system. In contrast, the association with WBC likely reflects systemic inflammatory activity. Our findings suggest that RBC and WBC counts—particularly within moderate ranges—are associated with the presence of myocardial infarction among cardiovascular patients at the time of hospital admission. However, the clinical utility of these hematological markers appears to be limited in the lowest and highest quartiles and requires further investigation. Importantly, our study confirms previously reported roles of WBC and RBC in the pathophysiology of MI, but distinguishes itself by analyzing these markers within a CVD patient population rather than among healthy controls. This provides novel insights into their relevance in a real-world clinical context.

The use of bootstrap validation not only confirmed the results obtained via classical statistical methods, but also improved the assessment of their stability and reduced the influence of sampling variability. Traditional parametric tests, although widely used, have limitations in small or highly skewed samples, which may lead to biased estimates and misleading conclusions. The bootstrap approach enabled more accurate estimation of the distribution of the test statistics, revealing subtle relationships that may be undetectable using conventional methods alone. Our findings highlight the value of resampling techniques in clinical research, where the stability and reliability of predictive models are essential for meaningful interpretation. The use of such methodology in future studies may enhance the robustness of statistical inference, improve result reproducibility, and enable more effective modeling of complex relationships in medical data.

## Figures and Tables

**Table 1:** Blood morphology, serum biochemistry, and chosen clinical, anthropometric, and sociological variables reported for the studied group.

Variable	Total Population	MI(−)	MI(+)	*p* Value
Age [years]	60.41 ± 15.14	59.65 ± 15.77	64.24 ± 10.69	0.02
Sex				0.02 ^C^
Male, *n* (%)	427 (57.47)	340 (54.84)	87 (70.73)	
Female, *n* (%)	316 (42.53)	280 (45.16)	36 (29.27)	
BMI [kg/m^2^]	27.20 ± 4.36	27.16 ± 4.37	27.36 ± 4.36	0.48
RBCs [10^6^/µL]	4.61 ± 0.51	4.62 ± 0.51	4.53 ± 0.49	0.19
WBCs [10^3^/µL]	7.94 ± 2.57	7.59 ± 2.31	9.7 ± 3.04	4.07 × 10^−9^
PLTs [10^3^/µL]	226.59 ± 60.12	225.35 ± 59.77	232.80 ± 61.77	0.31
PCT [%]	0.11 ± 0.06	0.24 ± 0.05	0.24 ± 0.06	0.28
PLCR [%]	29.16 ± 6.39	29.13 ± 6.4	29.3 ± 6.34	0.49
MPV [fL]	10.57 ± 0.92	10.57 ± 0.93	10.59 ± 0.92	0.49
Ht [%]	41.25 ± 4.20	41.31 ± 4.18	40.94 ± 4.26	0.39
Total cholesterol [mg/dL]	191.58 ± 48.06	191.64 ± 48.26	191.28 ± 47.26	0.51
LDL cholesterol [mg/dL]	114.83 ± 40.00	114.37 ± 39.92	117.15 ± 40.47	0.44
HDL cholesterol [mg/dL]	48.67 ± 15.04	49.55 ± 15.29	44.29 ± 12.92	0.01
Triglycerides [mg/dL]	143.48 ± 77.66	141 ± 72.14	155.94 ± 100.38	0.18
Glucose [mg/dL]	127.26 ± 44.44	123.39 ± 40.01	147.43 ± 58.87	5.82 × 10^−4^
HbA1c [%]	5.90 ± 0.88	5.88 ± 0.84	5.99 ± 1.01	0.39
hsCRP [mg/L]	17.49 ± 40.40	13.77 ± 36.36	35.89 ± 52.77	6.10 × 10^−4^

Variables (non-adjusted) are presented as means ± SD or as percentage fractions (%), with absolute counts (*n*) of the whole group of investigated patients. Data for the total population (*n* = 743), myocardial infarction (MI(+); *n* = 123), and non-myocardial infarction (MI(−); *n* = 620) groups. Comparisons between the MI(+) and MI(−) groups were performed using unpaired Student’s *t*-test for continuous variables and Chi-square test (^C^) for categorical variables. All *p*-values were estimated using bootstrap resampling (10,000 iterations). Abbreviations: BMI = body mass index, HbA1c = glycated hemoglobin, HDL = high-density lipoprotein, Ht = hematocrit, hsCRP = high-sensitivity C-reactive protein, LDL = low-density lipoprotein, MPV = mean platelet volume, PCT = plateletcrit, PLCR = platelet–large cell ratio, PLT = blood platelets, RBC = red blood cell, WBC = white blood cell.

**Table 2:** Basic characteristics of participants by quartiles of RBC value.

Variable	Quartiles				
	Q1 (*n* = 182)	Q2 (*n* = 186)	Q3 (*n* = 185)	Q4 (*n* = 190)	*p* Value
Age [years]	66.46 ± 13.52	61.88 ± 14.01	59.67 ± 14.57	53.92 ± 15.66	3.932 × 10^−8^
Sex					2.289 × 10^−10 C^
Male, *n* (%)	75 (41.21)	89 (47.85)	103 (55.68)	160 (84.21)	
Female, *n* (%)	107 (58.79)	97 (52.15)	82 (44.32)	30 (15.79)	
RBCs [10^6^/µL]	3.96 ± 0.30	4.45 ± 0.09	4.76 ± 0.10	5.24 ± 0.24	8.339 × 10^−253^
WBCs [10^3^/µL]	7.54 ± 2.67	7.68 ± 2.42	7.97 ± 2.46	8.54 ± 2.61	0.013
PLTs [10^3^/µL]	213.47 ± 61.83	225.67 ± 56.47	227.30 ± 56.82	239.34 ± 62.74	0.011
Myocardial infarction, *n* (%)					0.232 ^C^
Yes	35 (19.23)	30 (16.13)	34 (18.38)	24 (12.63)	
No	147 (80.77)	156 (83.87)	151 (81.62)	166 (87.37)	

Variables (non-adjusted) are presented as means ± SD or as percentage fractions (%), with absolute counts (*n*) for the quartile groups (Q1–Q4) based on RBC values. Comparisons between quartiles of RBC were performed using one-way ANOVA for continuous variables and Chi-square test (^C^) for categorical variables. All *p*-values were estimated using bootstrap resampling (10,000 iterations). Abbreviations: PLT = blood platelet, RBC = red blood cell, WBC = white blood cell.

**Table 3:** Basic characteristics of participants by quartiles of WBC value.

Variable	Quartiles				
	Q1 (*n* = 183)	Q2 (*n* = 187)	Q3 (*n* = 187)	Q4 (*n* = 186)	*p* Value
Age [years]	60.66 ± 16.53	61.80 ± 13.63	60.57 ± 14.18	58.62 ± 15.99	0.199
Sex					0.004 ^C^
Male, *n* (%)	87 (47.54)	96 (51.34)	118 (63.10)	126 (67.74)	
Female, *n* (%)	96 (52.46)	91 (48.66)	69 (36.90)	60 (32.26)	
RBCs [10^6^/µL]	4.50 ± 0.50	4.61 ± 0.48	4.62 ± 0.52	4.70 ± 0.52	0.019
WBCs [10^3^/µL]	5.29 ± 0.71	6.73 ± 0.34	8.24 ± 0.57	11.45 ± 2.14	4.079 × 10^−225^
PLTs [10^3^/µL]	203.66 ± 51.15	217.69 ± 50.52	231.64 ± 57.76	253.00 ± 68.39	3.636 × 10^−9^
Myocardial infarction, *n* (%)					4.130 × 10^−7 C^
Yes	10 (5.46)	22 (11.76)	26 (13.90)	65 (34.95)	
No	173 (94.54)	165 (88.24)	161 (86.10)	121 (65.05)	

Variables (non-adjusted) are presented as means ± SD or as percentage fractions (%), with absolute counts (*n*) for the quartile groups (Q1–Q4) based on WBC values. Comparisons between quartiles of WBC were performed using one-way ANOVA for continuous variables and Chi-square test (^C^) for categorical variables. All *p*-values were estimated using bootstrap resampling (10,000 iterations). Abbreviations: PLT = blood platelet, RBC = red blood cell, WBC = white blood cell.

## Data Availability

Data supporting the reported results can be made available upon request. For details on how to access the data, please contact the corresponding author.

## Supplementary Materials

The following supporting information can be downloaded at: https://www.mdpi.com/article/10.3390/ijms26125736/s1.

## Abbreviations

The following abbreviations are used in this manuscript:

AMI	Acute Myocardial Infarction
ATP	Adenosine Triphosphate
BMI	Body Mass Index
CVD	Cardiovascular Disease
cGMP	Cyclic Guanosine Monophosphate
HbA1c	Glycated Hemoglobin
HDL	High-Density Lipoprotein
Ht	Hematocrit
hsCRP	High-Sensitivity C-Reactive Protein
LDL	Low-Density Lipoprotein
MI	Myocardial Infarction
MPV	Mean Platelet Volume
NO	Nitric Oxide
OR	Odds Ratio
PCT	Plateletcrit
PLCR	Platelet–Large Cell Ratio
PLT	Platelets/Blood Platelets
Q1	First Quartile
Q2	Second Quartile
Q3	Third Quartile
Q4	Fourth Quartile
RBC	Red Blood Cell
STEMI	ST-Elevation Myocardial Infarction
WBC	White Blood Cell
