# Supplementary material for: Red Blood Cell, White Blood Cell, and Platelet Counts as Differentiating Factors in Cardiovascular Patients with and Without Current Myocardial Infarction
Source: Int J Mol Sci. 2025 Jun 15;26(12):5736. doi: 10.3390/ijms26125736 (PMC12192950; doi:10.3390/ijms26125736)
Supplement: Supplementary file 1 [file ijms-26-05736-s001.zip › Table S1.pdf]

Table S1. Clinical characteristics of the study population, including the prevalence of cardiovascular diseases and comorbid conditions.

Variable	Total population	MI(+)	MI(-)
Myocardial infarction			
Yes	16.55 (123)		
No	83.45 (620)		
Diabetes Mellitus			
Yes	21.80 (162)	24.39 (30)	21.29 (132)
No	77.93 (579)	75.61 (93)	78.39 (486)
Spontaneous Coronary Artery Dissection			
Yes	29.07 (216)	46.34 (57)	25.65 (159)
No	70.12 (521)	53.66 (66)	73.39 (455)
Congenital Heart Defect			
Yes	6.33 (47)	3.25 (4)	6.94 (43)
No	93.67 (696)	96.75 (119)	93.06 (577)
Acquired Heart Defect			
Yes	53.03 (394)	52.85 (65)	53.06 (329)
No	46.84 (348)	47.15 (58)	46.77 (290)
Myocarditis			
Yes	8.21 (61)	3.25 (4)	9.19 (57)
No	91.79 (682)	96.75 (119)	90.81 (563)
Hypertension			
Yes	68.24 (507)	73.17 (90)	67.26 (417)
No	31.09 (231)	26.83 (33)	31.94 (198)
Congestive Heart Failure			
Yes	24.36 (181)	26.83 (33)	23.87 (148)
No	74.16 (551)	72.36 (89)	74.52 (462)
Arrhythmias			
Yes	46.70 (347)	24.39 (30)	51.13 (317)
No	53.30 (396)	75.61 (93)	48.87 (303)
Peripheral Artery Disease (Lower Limbs)			
Yes	5.38 (40)	8.13 (10)	4.84 (30)
No	92.19 (685)	87.8 (108)	93.06 (577)
Carotid Artery Atherosclerosis			
Yes	9.56 (71)	13.82 (17)	8.71 (54)
No	86.68 (644)	82.93 (102)	87.42 (542)
Venous Thrombosis (Lower Limbs)			
Yes	5.38 (40)	3.25 (4)	5.81 (36)
No	94.48 (702)	96.75 (119)	94.03 (583)
Pulmonary Embolism			
Yes	2.96 (22)	0.81 (1)	3.39 (21)
No	96.77 (719)	98.37 (121)	96.45 (598)

Variables (non-adjusted) are presented as counts and percentages % (n) for the total cohort (N = 743) and for the MI+ and MI− groups.
