# Supplementary material for: Red Blood Cell, White Blood Cell, and Platelet Counts as Differentiating Factors in Cardiovascular Patients with and Without Current Myocardial Infarction
Source: Int J Mol Sci. 2025 Jun 15;26(12):5736. doi: 10.3390/ijms26125736 (PMC12192950; doi:10.3390/ijms26125736)
Supplement: Supplementary file 1 [file ijms-26-05736-s001.zip › Table S2.pdf]

Table S2. Association between myocardial infarction and peripheral blood cell counts (RBC, WBC, PLT) in patients with cardiovascular disease: analysis in the total study population and stratified by STEMI and NSTEMI subtypes.

		Classical approach			Bootstrap approach		
		OR	95% CI	p-value	OR	95% CI	p-value
All patients							
	Age	1.028	1.012-1.045	**0.001**	1.034	1.020-1.048	**4.214×10^{-4}**
	Sex	1.926	1.211-3.064	**0.006**	1.975	1.376-2.834	**0.011**
	RBC	0.637	0.410-0.988	**0.044**	0.609	0.425-0.872	0.050
	WBC	1.344	1.239-1.458	**1.137×10^{-12}**	1.382	1.284-1.488	**4.983×10^{-12}**
	PLT	1.000	0.997-1.004	0.871	1.000	0.998-1.003	0.479
STEMI							
	Age	1.012	0.986–1.039	0.359	1.019	1.004–1.035	0.075
	Sex	2.263	0.944–5.426	0.067	3.603	2.266–5.730	**8.56×10^{-5}**
	RBC	0.666	0.318–1.393	0.280	0.501	0.328–0.766	**0.023**
	WBC	1.482	1.309–1.678	**5.47×10^{-10}**	1.629	1.501–1.767	**5.43×10^{-25}**
	PLT	1.001	0.996–1.007	0.634	1.004	1.001–1.008	0.089
NSTEMI							
	Age	1.026	1.003–1.049	**0.028**	1.034	1.021–1.048	**2.05×10^{-4}**
	Sex	1.790	0.951–3.371	0.071	1.809	1.286–2.544	**0.019**
	RBC	0.646	0.366–1.139	0.131	0.602	0.437–0.829	**0.025**
	WBC	1.222	1.095–1.365	**3.48×10^{-4}**	1.285	1.192–1.385	**1.06×10^{-6}**
	PLT	1.000	0.995–1.004	0.848	0.999	0.996–1.002	0.399

The results of the analysis are presented as odds ratios (OR) with confidence intervals, calculated with or without the use of the bootstrap resampling procedure (10000 iterations). In the bootstrap approach OR values were adjusted to equal sample sizes of 372 in both the MI+ and MI− groups. The odds ratios were computed for the entire patient group (N = 743). The p-value for the Hosmer-Lemeshow test exceeded 0.05. Abbreviations: PLT = blood platelets, RBC = red blood cells, WBC = white blood cells.
