# Supplementary material for: Red Blood Cell, White Blood Cell, and Platelet Counts as Differentiating Factors in Cardiovascular Patients with and Without Current Myocardial Infarction
Source: Int J Mol Sci. 2025 Jun 15;26(12):5736. doi: 10.3390/ijms26125736 (PMC12192950; doi:10.3390/ijms26125736)
Supplement: Supplementary file 1 [file ijms-26-05736-s001.zip › Table S4.pdf]

Table S4. Association between myocardial infarction and blood cell counts in patients with cardiovascular disease. Separate models were constructed for each platelet-related index (PLT, PCT, PLCR, MPV), while RBC and WBC were consistently included. Analyses were conducted in the total study population. All models were adjusted for age and sex.

	Classical approach			Bootstrap approach		
	OR	95% CI	p-value	OR	95% CI	p-value
Age	1.028	1.012–1.046	**0.001**	1.034	1.020–1.049	**3.507×10^{-4}**
Sex	1.905	1.191–3.046	**0.007**	1.923	1.333–2.775	**0.016**
RBC	0.634	0.407–0.987	**0.044**	0.611	0.425–0.876	0.052
WBC	1.347	1.239–1.464	**2.384×10^{-12}**	1.389	1.288–1.499	**8.372×10^{-12}**
PCT	1.099	0.022–55.845	0.962	4.045	0.152–109.554	0.500
Age	1.028	1.011–1.045	**0.001**	1.034	1.020–1.048	**3.80×10^{-4}**
Sex	1.928	1.218–3.050	**0.005**	1.960	1.375–2.794	**0.010**
RBC	0.637	0.410–0.989	**0.044**	0.609	0.424–0.873	0.049
WBC	1.344	1.243–1.453	**1.299×10^{-13}**	1.385	1.290–1.486	**1.367×10^{-13}**
PLCR	0.998	0.966–1.031	0.904	0.995	0.970–1.020	0.467
Age	1.029	1.012–1.046	**0.001**	1.034	1.020–1.049	**2.798×10^{-4}**
Sex	1.896	1.197–3.003	**0.006**	1.922	1.348–2.742	**0.012**
RBC	0.634	0.408–0.985	**0.043**	0.605	0.422–0.868	**0.047**
WBC	1.349	1.247–1.459	**8.127×10^{-14}**	1.391	1.295–1.493	**1.10×10^{-13}**
MPV	0.98	0.783–1.228	0.862	0.963	0.808–1.147	0.459

The results of the analysis are presented as odds ratios (OR) with confidence intervals, calculated with or without the use of the bootstrap resampling procedure (10000 iterations). In the bootstrap approach OR values were adjusted to equal sample sizes of 372 in both the MI+ and MI− groups. The odds ratios were computed for the entire patient group (N = 743). The p-value for the Hosmer-Lemeshow test exceeded 0.05. Abbreviations: MPV = mean platelet volume, PCT= plateletcrit, PLCR = platelet large cell ratio, PLT = blood platelets, RBC = red blood cells, WBC = white blood cells.
