# Supplementary material for: Red Blood Cell, White Blood Cell, and Platelet Counts as Differentiating Factors in Cardiovascular Patients with and Without Current Myocardial Infarction
Source: Int J Mol Sci. 2025 Jun 15;26(12):5736. doi: 10.3390/ijms26125736 (PMC12192950; doi:10.3390/ijms26125736)
Supplement: Supplementary file 1 [file ijms-26-05736-s001.zip › Table S5.pdf]

Table S5 Association between myocardial infarction and the numbers of red blood cells and white blood cells in cardiovascular patients with varying RBC levels.

Model I		Classical approach			Bootstrap approach		
		OR	95% CI	p-value	OR	95% CI	p-value
Q1 (N=182)							
	Age	1.021	0.990-1.052	0.188	1.023	1.011-1.036	**0.008**
	Sex	1.869	0.880-3.967	0.104	1.889	1.403-2.544	**0.004**
	RBC	1.484	0.403-5.472	0.553	1.455	0.878-2.416	0.296
Q2 (N=186)							
	Age	1.008	0.976-1.041	0.630	1.007	0.993-1.021	0.362
	Sex	6.633	2.477-17.762	**1.667×10^{-4}**	7.967	5.484-11.577	**8.548×10^{-20}**
	RBC	0.413×10^{-3}	3.113×10^{-6}-0.055	**1.779×10^{-3}**	4.205×10^{-4}	6.762×10^{-5}-2.627×10^{-3}	**3.197×10^{-9}**
Q3 (N=185)							
	Age	1.045	1.010-1.082	**0.012**	1.052	1.037-1.066	**2.474×10^{-8}**
	Sex	1.663	0.747-3.702	0.213	1.624	1.187-2.223	**0.036**
	RBC	0.075	0.001-4.427	0.213	0.114	0.024-0.533	**0.026**
Q4 (N=190)							
	Age	1.018	0.988-1.049	0.244	1.022	1.010-1.033	**0.008**
	Sex	5.950	0.750-47.213	0.091	6.677	3.582-12.510	**2.410×10^{-6}**
	RBC	0.994	0.156-6.320	0.995	0.845	0.436-1.638	0.435

Model II		Classical approach			Bootstrap approach		
		OR	95% CI	p-value	OR	95% CI	p-value
Q1 (N=182)							
	Age	1.021	0.990-1.052	0.186	1.024	1.011-1.038	**0.01**
	Sex	1.52	0.684-3.378	0.305	1.512	1.099-2.081	0.077
	RBC	1.711	0.448-6.536	0.433	1.507	0.882-2.577	0.293
	WBC	1.279	1.112-1.471	**0.001**	1.357	1.268-1.453	**2.56×10^{-11}**
Q2 (N=186)							
	Age	1.028	0.988-1.069	0.170	1.032	1.014-1.050	**0.013**
	Sex	4.166	1.422-12.200	**0.009**	4.114	2.735-6.190	**1.552×10^{-6}**
	RBC	5.334×10^{-5}	1.394×10^{-7}-2.04×10^{-2}	**0.001**	5.192×10^{-5}	6.252×10^{-6}-4.346×10^{-4}	**9.206×10^{-14}**
	WBC	1.578	1.289-1.933	**1.000×10^{-5}**	1.599	1.453-1.759	**3.901×10^{-19}**
Q3 (N=185)							
	Age	1.053	1.015-1.091	**0.006**	1.059	1.044-1.075	**9.866×10^{-10}**
	Sex	1.670	0.733-3.806	0.222	1.577	1.136-2.189	0.060
	RBC	0.068	0.001-4.466	0.208	0.090	0.018-0.468	**0.019**
	WBC	1.286	1.095-1.511	**0.002**	1.280	1.196-1.369	**5.507×10^{-9}**
Q4 (N=190)							
	Age	1.021	0.988-1.055	0.221	1.025	1.011-1.038	**0.008**
	Sex	5.288	0.615-45.474	0.129	5.022	2.563-9.910	**3.392×10^{-4}**
	RBC	1.206	0.163-8.911	0.854	1.517	0.735-3.132	0.376
	WBC	1.394	1.184-1.642	**6.856×10^{-5}**	1.508	1.398-1.627	**2.101×10^{-19}**

The results of the analysis are presented as odds ratios (OR) with confidence intervals, calculated with or without the use of the bootstrap resampling procedure (10000 iterations). OR values were adjusted to equal sample sizes of 372 in both the MI+ and MI− groups. The analysis was performed across

three distinct models to investigate the influence of selected variables on the myocardial infarction. Model I examined the effects of age, sex, and RBC. Model II, in addition to age and sex, included RBC, and WBC—allowing for the assessment of their collective impact on the outcome. The odds ratios were computed for the entire patient group, stratified based on quartiles of RBC values. In all models, the p-values for the Hosmer-Lemeshow test were greater than 0.05. Abbreviations: RBC = red blood cells, WBC = white blood cells.
