# Supplementary material for: Red Blood Cell, White Blood Cell, and Platelet Counts as Differentiating Factors in Cardiovascular Patients with and Without Current Myocardial Infarction
Source: Int J Mol Sci. 2025 Jun 15;26(12):5736. doi: 10.3390/ijms26125736 (PMC12192950; doi:10.3390/ijms26125736)
Supplement: Supplementary file 1 [file ijms-26-05736-s001.zip › Table S6.pdf]

Table S6 Association between myocardial infarction and the numbers of red blood cells and white blood cells in cardiovascular patients with varying WBC levels.

Model I		Classical approach			Bootstrap approach		
		OR	95% CI	p-value	OR	95% CI	p-value
Q1 (N=183)							
	Age	1.054	0.999-1.113	0.057	1.076	1.059-1.093	**4.731×10^{-16}**
	Sex	1.000	0.260-3.839	1.000	1.015	0.722-1.427	0.494
	WBC	0.797	0.341-1.859	0.599	0.887	0.731-1.076	0.302
Q2 (N=187)							
	Age	1.017	0.982-1.054	0.348	1.022	1.009-1.036	**0.015**
	Sex	1.403	0.562-3.503	0.468	1.445	1.076-1.94	0.090
	WBC	1.745	0.459-6.633	0.414	2.197	1.363-3.544	**0.026**
Q3 (N=187)							
	Age	1.047	1.009-1.086	**0.015**	1.055	1.040-1.070	**3.070×10^{-9}**
	Sex	1.806	0.698-4.674	0.223	2.105	1.505-2.946	**0.002**
	WBC	1.492	0.701-3.175	0.299	1.610	1.225-2.117	**0.017**
Q4 (N=186)							
	Age	1.032	1.009-1.054	**0.005**	1.034	1.023-1.046	**3.426×10^{-6}**
	Sex	2.495	1.195-5.212	0.015	2.582	1.822-3.660	**1.163×10^{-4}**
	WBC	1.161	1.004-1.344	**0.044**	1.169	1.086-1.258	**0.003**

Model II		Classical approach			Bootstrap approach		
		OR	95% CI	p-value	OR	95% CI	p-value
Q1 (N=183)							
	Age	1.054	0.997-1.115	0.066	1.074	1.057-1.091	**2.646×10^{-14}**
	Sex	1.000	0.259-3.857	1.000	1.055	0.745-1.493	0.488
	RBC	1.005	0.247-4.086	0.995	0.805	0.523-1.238	0.337
	WBC	0.796	0.334-1.900	0.607	0.903	0.741-1.100	0.353
Q2 (N=187)							
	Age	1.011	0.974-1.050	0.571	1.016	1.002-1.030	0.093
	Sex	1.621	0.624-4.208	0.321	1.690	1.238-2.308	**0.021**
	RBC	0.570	0.201-1.616	0.291	0.559	0.398-0.786	**0.015**
	WBC	1.780	0.469-6.756	0.397	2.425	1.491-3.945	**0.014**
Q3 (N=187)							
	Age	1.035	0.996-1.075	0.076	1.047	1.032-1.063	**2.490×10^{-6}**
	Sex	2.277	0.849-6.109	0.102	3.029	2.102-4.365	**2.322×10^{-5}**
	RBC	0.369	0.155-0.881	**0.025**	0.288	0.199-0.418	**8.541×10^{-7}**
	WBC	1.574	0.726-3.416	0.251	1.623	1.224-2.153	**0.019**
Q4 (N=186)							
	Age	1.028	1.005-1.052	**0.018**	1.031	1.019-1.043	**1.486×10^{-4}**
	Sex	2.606	1.238-5.486	**0.012**	2.752	1.928-3.927	**5.310×10^{-5}**
	RBC	0.756	0.390-1.466	0.408	0.748	0.536-1.045	0.195
	WBC	1.154	0.996-1.338	0.056	1.162	1.079-1.251	**0.005**

The results of the analysis are presented as odds ratios (OR) with confidence intervals, calculated with or without the use of the bootstrap resampling procedure (10000 iterations). OR values were adjusted to equal sample sizes of 372 in both the MI+ and MI− groups. The analysis was performed across three distinct models to investigate the influence of selected variables on the myocardial infarction. Model I examined the effects of age, sex, and WBC. Model II, in addition to age and sex, included RBC, and WBC—allowing for the assessment of their collective impact on the outcome. The odds ratios were computed for the entire patient group, stratified based on quartiles of WBC values. In all models, the p-values for the Hosmer-Lemeshow test were greater than 0.05. Abbreviations: RBC = red blood cells, WBC = white blood cells.
